# Eradication of a chronic wound and driveline infection after redo-LVAD implantation

**DOI:** 10.1186/1749-8090-9-63

**Published:** 2014-03-31

**Authors:** Veronika Walter, Ulrich A Stock, Mauricio Soriano-Romero, Andreas Schnitzbauer, Anton Moritz, Andres Beiras-Fernandez

**Affiliations:** 1 Department Thoracic & Cardiovascular Surgery, Johann Wolfgang Goethe University Hospital, Frankfurt am Main, Theodor-Stern-Kai 7, Frankfurt 61590, Germany; 2 Department General Surgery, Johann Wolfgang Goethe University Hospital, Frankfurt am Main, Frankfurt, Germany

**Keywords:** Daptomycin, LVAD, MRSA, Infection eradication

## Abstract

A 48 year old patient with dilated cardiomyopathy and chronic acne inversa underwent implantation of a LVAD system (Heartmate II, Thoratec, USA) March 2011. During 2011 and 2012 the patient was repeatedly readmitted for treatment of driveline infection with MRSA. Colonization was controlled with Linezolid and Rifampicin however reoccurred after discontinuation. In August 2012 the LVAD-system was exchanged due to pump dysfunction (HVAD, HeartWare Inc., USA). Postoperatively, the patient presented with ascites which secreted through the driveline exit. Consequently, the abdominal wall was surgically corrected to prevent exit of peritoneal fluid through the driveline, and the patient was discharged with sterile wound swabs. However 6 weeks after discharge the driveline exit wound started secreting pus showing abundant growth of multi resistant staphylococcus aureus (MRSA). With clinical signs of increasing liver failure with regular need for paracentesis, and clinical signs of local infection, a CT scan of the abdomen was performed revealing an enrichment of contrast medium along the driveline and an abscess-like formation on the abdominal wall. Patient was admitted receiving regular dose Daptomycin and Rifampicin. The latter was discontinued after ten days. The abscess, surrounding driveline exit and abdominal wall cavity was excised and vacuum treatment initiated. Total duration of Daptomycin therapy was 3 weeks. While first week skin and wound swabs were still positive for MRSA, all samples were sterile after the second week. Inflammation was monitored by leucocyte count and IL6. The secretion of pus along the driveline ceased, the wound cavity was closed subsequently. After discharge and stop of antibiotics skin and driveline swabs remained negative for MRSA (10 weeks).

## Background

Left ventricular assist device (LVAD) implantation is not only a bridge-to-transplantation option for patients awaiting a donor heart, but is often used as bridge-to-destination therapy in patients unsuitable for transplantation for various reasons. Device infection remains a threatening complication, which may lead to prolonged hospitalisation, need to device exchange, urgent transplantation, and even death of the patient [1]. Infections with multidrug resistant (MDR) organisms pose major difficulties for eradication therapy. Especially patients who are subject to continuous hospital treatments risk contamination or change of resistances spectrum. Gold standard therapy of certain organisms often fails to eradicate surface associated colonisations of implanted devices such as cardiovascular implants, while failed eradication leads to numerous complications and an increased mortality rate among the affected patients. Device infections through multidrug resistant bacteria, such as MRSA, are often resistant even to first-line antibiotics, due to extended resistance spectrum and reduce tissue penetration in scar tissue after multiple surgical procedures. In addition a solid biofilm on devices is often impenetrable even for suitable antibiotics because of the isolating nature of biofilms [1-3].

Daptomycin is a cyclic lipopeptide with excellent bactericidal efficacy especially upon infections of skin and soft tissue where last resort antibiotics like Linezolid and Vancomycin fail. A synergistic action of Rifampicin and Daptomycin for the treatment of MDR organisms has been previously reported [4].

We report about a patient with dilated cardiomyopathy who 6 months after redo implantation of a LVAD presenting with a chronic driveline infection with MRSA. This patient was treated successfully with a combination of surgical wound management and antibiotic treatment based on Daptomycin and Rifampicin.

## Case presentation

In March 2011 a 48 year old patient (male, caucasian) with dilated cardiomyopathy, congestive heart failure, chronic liver and kidney failure, and a chronic condition of acne inversa underwent an implantation of an LVAD system (Heartmate II, Thoratec, USA) due to rapid cardiopulmonary deterioration. At the time of the implantation, the velour surrounding the driveline was partly exteriorized to provide better connection with the surrounding tissue. Initially a right ventricular assist device (RVAD) was implanted in extra corporal oxygenation mode (ECMO). After cardiopulmonary stabilisation the ECMO was explanted on postoperative day 6. The patient was discharged into rehabilitation after further clinical stabilisation. During 2011 and 2012 the patient was repeatedly readmitted for treatment of driveline infections with MRSA. Initially treated with Linezolid and Rifampicin as antibiotic therapy, the colonization could only be controlled under antibiotic therapy and reoccurred after discharge. Skin and surface swabs of the VAD system showed growth of MRSA between clinical stays. With this patient, the LVAD therapy was considered a bridge-to-destination therapy, both because of lack of compliance regarding smoking and nutrition and because of a severe cirrhosis cardiaque possibly combined with nutritive liver cirrhosis that made a simultaneous liver transplantation necessary.

In August 2012 a rapid deterioration of the patient’s health condition led to the diagnosis of LVAD dysfunction with bacterial superinfection of the pump system and severe pneumonia. At admission anticoagulation was sufficient, even slightly above the INR ratio requested for LVAD function because of ongoing liver failure. Nevertheless a DIC (disseminated intravascular coagulation) associated with the septic state might have led to clot formation inside the VAD system. A solid bacterial biofilm inside the LVAD pump was discussed a reason for system failure.

Listing for high urgent transplantation was dismissed. Not only there was a need to perform a combined heart and liver transplantation because of acute-on-chronic liver failure but also the highly unstable cardiopulmonary situation of the patient with multi organ failure did not permit such an extensive operation by means of a possible negative outcome on postoperative morbidity and mortality.

Because of therapy resistant output failure despite increasing doses of catecholaminergic support an arterio-venous ECMO was implanted prior to redo surgery. To assess cardiac function before redo surgery numerous transoesophageal echocardiograms were conducted, showing a global strongly impaired ventricular function and insufficiency of the AV valves, but no sign of endocarditis. After implantation of a new LVAD system (HVAD; HeartWare Inc., USA) and prolonged stay in the intensive care unit the patient recovered. Implantation of a HeartWare LVAD system was chosen because of a thinner driveline with a shorter velour sleeve that does not exit the wound. Also the patient had suffered from chronic left sided thoracic pain located at the site of the former LVAD pump. Because of the pump size and its position in the thoracic cavity the pump was always in very close vicinity to the ribcage, almost penetrating through the intercostal muscle layer. A better fit could be achieved at redo implantation with the smaller HeartWare pump.

The presumably infected LVAD system was completely removed, including extirpation of the velour sheet around the driveline and extensive surgical debridement of the surrounding scarred subcutaneous tissue.

During his stay, the patient developed ascites with a need for regular paracentesis (once per week). Secretion of peritoneal fluid through the driveline exit wound was observed. A surgical intervention to tighten the exit channel of the driveline was successfully performed. The patient was discharged into ambulatory treatment on day 35 following redo surgery.

While wound swabs during the hospitalisation period showed no bacterial growth even after discontinuation of antibiotics soon after discharge the driveline exit wound started secreting pus. All wound swabs showed abundant growth of MRSA, but also regular skin swabs showed growth of MRSA. In order to find a possible source for reinfection with MRSA household members of the patient were checked for MRSA, but with no positive result.

**Figure 1:**
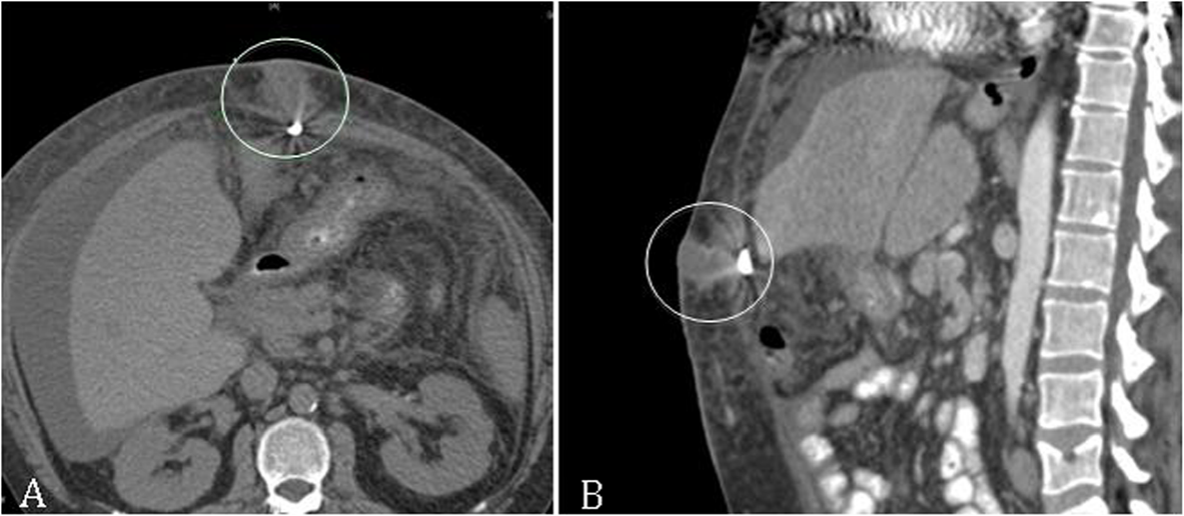
CT-scan of abdominal abscess formation (circles), A-Axial view; B-Sagittal view.

Because of increasing liver failure with regular need for ambulatory paracentesis as well a local signs of infection a CT scan of the abdomen was performed. The CT scan showed an enrichment of contrast medium along the driveline, especially in the superficial parts close to the exit wound as well as an abscess-like formation on the abdominal wall. A sample that was obtained from this formation by ultrasound-guided puncture was also positive for MRSA (Figure 1). The patient was readmitted to receive an antibiotic regime of intravenous (i.v.) Daptomycin and Rifamipcin, both applied in regular dose. Rifampicin was discontinued ten days after initiation of therapy due to the pre-existent liver dysfunction of the patient. Additionally surgical exstirpaton of the abscess and the tissue surrounding the driveline exit were performed, including all parts of velour that were accessible to operative debridement. Vacuum wound treatment (VAC system, KCI, USA) was applied in the remaining wound cavity on the site of the excised abscess.

**Figure 2:**
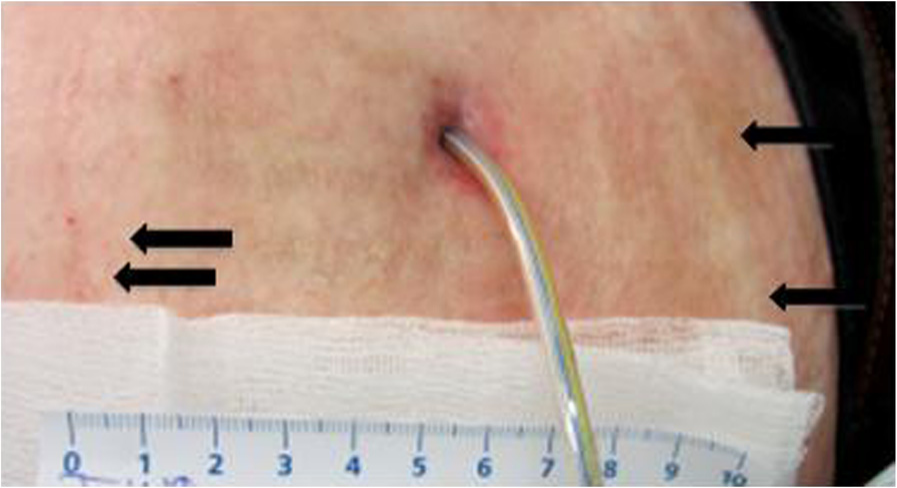
**Skin condition following treatment.** Notice normal scar formation (arrows) without any signs of inflammation.

The total duration of Daptomycin therapy exceeded 3 weeks (24 days). During the first week skin and wound swabs were still positive for MRSA however after the second week all samples remained sterile. All samples were obtained under antibiotic therapy during the hospital stay. Inflammation parameters were monitored through leucocyte count and Interleukine 6 (IL6). Secretion of pus along the driveline was never observed after operative debridement, the remaining abscess cavity was closed subsequently. Leucocyte count and IL6 returned to normal values. After discharge and discontinuation of antibiotic therapy skin and driveline swabs remained negative for MRSA (Figure 2). The patient had to appear weekly at the outpatient clinic of the cardiothoracic department. At each visit blood samples, skin and wound swabs were obtained, and a clinical examination with focus on the wound condition was performed. The patient remained negative for MRSA at 6 months follow-up. For follow-up of the cardiac condition ultrasound examinations were performed regularly during the entire follow-up.

## Discussion

Device infections with MDR organisms still pose a major threat, with alternatives for surgical treatment being limited to device exchange of local debridement in less severe cases. Device exchange however has a high rate of mortality, as the patients in need of device exchange are often subject to multiple comorbidities and usually in a septic or sub-septic state at time of device exchange [1,4].

As with all implanted cardiac devices, device endocarditis is considered to be one of the most challenging complications. While conservative treatment of a colonized LVAD system may be successful, focus removal of infected valves becomes far more complicated when after failed conservative treatment neither valve function nor organ function can be restored without surgical intervention. Floating vegetations can also lead to embolisation and pump dysfunction when sucked into the inflow cannula of the VAD system, apart from leading to organ embolisation. Therefore it is vital to perform thorough echocardiogram examinations on patients in risk of LVAD endocarditis when device infection is suspected.

Antibiotic treatment of MRSA infections proved to become increasingly difficult, with strains of MRSA with intermediate efficacy for Vancomycin and other last resort antibiotics, or even fully resistant strains [3]. For these highly specific infections Daptomycin has proved to be an effective treatment [1,4,5]. To prevent prolonged hospitalisation and need for device exchange, we recommend an aggressive antibiotic therapy with Daptomycin combined with local debridement and vacuum treatment in infected wound cavities. In addition to this, after patient discharge, regular follow-up visits with trained staff familiar with LVAD treatment are necessary to detect reoccurrence of any infection. Prolonged antibiotic therapy can be applied in cases without local tissue complications and should be treated as aggressive as a non-native-valve endocarditis, preferably in cooperation with the infectiology department.

In this case we conclude that i.v. application of Daptomycin is a sufficient and effective therapy in eradication of colonization with multidrug-resistant organisms. There have been several cases of successfully eradicated MRSA or other MDR-organism infected patients, yet all cases remain single-hospital/single-site experiences. A collection of similar cases in one hospital should be obtained to gain further insight of the dynamics involved in eradication of MDR organisms with Daptomycin.

## Conclusions

In the present case we showed that successful eradication of a chronic LVAD driveline infection with MRSA in our patient was only possible when approached both surgically and conservatively. It is important to point out that while this was a single case study, conclusions towards broader applications can still be drawn from it. Especially in patients with several comorbidities that suffer from chronic infections of non-native material, a similar aggressive approach for eradication can be attempted in order to avoid even more aggressive redo operations.

## Consent

Written and informed consent was obtained from the patient for publication of this case report and any accompanying images. A copy of the written consent is available for review by the Editor-in-Chief of this journal.

## Abbreviations

LVAD: Left ventricular assist device; MRSA: Methicillin resistant Staphylococcus aureus; IL6: Interleukine 6; MDR: Multidrug-resistant; DIC: Disseminated intravascular coagulation; ECMO: Extracorporal membrane oxygenation; CT: Computer tomography; VISA: Vancomycin intermediate Staphylococcus aureus.

## Competing interests

AB-F received lecture fees from Novartis Pharma. The other authors declare that they have no competing interests.

## Authors’ contributions

VW was involved in all parts of the stationary treatment of the patient and in the postoperative wound management and VAC therapy. MS-R, AS and AB-F performed the debridement operation. All authors read and approved the final manuscript.
